# A minimum data standard for vector competence experiments

**DOI:** 10.1038/s41597-022-01741-4

**Published:** 2022-10-19

**Authors:** Velen Yifei Wu, Binqi Chen, Rebecca Christofferson, Gregory Ebel, Anna C. Fagre, Emily N. Gallichotte, Amy R. Sweeny, Colin J. Carlson, Sadie J. Ryan

**Affiliations:** 1 Center for Global Health Science and Security, Georgetown University Medical Center, Washington, D.C., USA; 2 Department of Pathobiological Sciences, Louisiana State University, Baton Rouge, USA; 3 Center for Vector-borne Infectious Diseases, Colorado State University, Fort Collins, USA; 4 School of Biosciences, University of Sheffield, Sheffield, United Kingdom; 5 Department of Microbiology and Immunology, Georgetown University Medical Center, Washington, D.C., USA; 6 Department of Biology, Georgetown University, Washington, USA; 7 Department of Geography, University of Florida, Gainesville, USA; 8 Emerging Pathogens Institute, University of Florida, Gainesville, USA; 9 College of Life Sciences, University of KwaZulu Natal, Durban, South Africa

**Keywords:** Viral transmission, Model invertebrates

## Abstract

The growing threat of vector-borne diseases, highlighted by recent epidemics, has prompted increased focus on the fundamental biology of vector-virus interactions. To this end, experiments are often the most reliable way to measure vector competence (the potential for arthropod vectors to transmit certain pathogens). Data from these experiments are critical to understand outbreak risk, but – despite having been collected and reported for a large range of vector-pathogen combinations – terminology is inconsistent, records are scattered across studies, and the accompanying publications often share data with insufficient detail for reuse or synthesis. Here, we present a minimum data and metadata standard for reporting the results of vector competence experiments. Our reporting checklist strikes a balance between completeness and labor-intensiveness, with the goal of making these important experimental data easier to find and reuse in the future, without much added effort for the scientists generating the data. To illustrate the standard, we provide an example that reproduces results from a study of *Aedes aegypti* vector competence for Zika virus.

## Introduction

Vector competence is an arthropod vector’s ability to transmit a pathogen after exposure to the pathogen^1–3^. It combines the intrinsic potential of a pathogen to successfully enter and replicate within the vector, and then disseminate to, replicate within, and release from the vector’s salivary glands into the saliva at sufficiently high concentration to initiate infection in the next vertebrate host. Quantifying this process at each step within the vector is fundamental to understanding and predicting vector-borne disease transmission.

Due to the inherent complexity of arboviral transmission, experimental studies of vector competence are also necessarily complex, and may report a number of types of data. Experimental settings add additional constraints, as controlled laboratory conditions are themselves inherently complex, and vector competence is highly responsive to some of these conditions (e.g., the temperature at which experiments take place). While the complexity and requisite scientific skills make these experiments challenging, their importance and value – particularly in response to vector-borne disease outbreaks of international concern – cannot be overstated, and has led to increasing numbers of these experiments. However, the complexity of the experiments, and the variety of conditions under which they are conducted, make it difficult to meticulously share (and synthesize) all relevant metadata, especially with consistent enough terminology to compare results across studies^4^. Because primary data are not reported in a standardized manner, opportunities are being lost to advance science and public health.

Here, we propose a minimum data standard for reporting the results of vector competence experiments. The motivation to create and disseminate data standards for reporting is part of a broad effort across scientific disciplines to preserve data for future use, recover existing data that may be unsearchable for many reasons, and establish open principles for harmonizing those data to better leverage the effort of the larger community of research^5–10^. In particular, the FAIR (Findability, Accessibility, Interoperability, and Reusability) guiding principles^11,12^ were created to improve the infrastructure supporting the reuse of scholarly data, including public data archiving^13^. These principles aim to maximize the value of research investments and digital publishing, and have been adopted into both efforts to synthesize and populate databases for use by the scientific community, and into the language of a growing number of funders’ reporting requirements. Tailoring FAIR principles to different subfields of scientific research requires consideration of the specific kinds of data that are regularly generated, and how they would best be reported. For example, the recently published minimum data standard MIReAD (Minimum Information for Reusable Arthropod abundance Data) aims to improve the transparency and reusability of arthropod abundance data^14^, thereby improving the benefits reaped from data sharing, and reducing the cost of obtaining research results. Importantly, these data standards do not aim to provide guidance on how experiments are conducted, nor guide research, but provide a reporting standard flexible enough to accommodate the outputs of most of these experiments.

In this paper, we characterize the key steps of vector competence experiments, and the data generated at each stage, as a means to establish common guidelines for data reporting that follow FAIR principles. Due to the long history of experimental work with mosquito vectors (and the incomparable role it plays in efforts to decrease the global burden of vector-borne disease), we propose a minimum data standard focused on capturing results from studies that test pairs of mosquitoes and arboviruses. However, we intentionally aimed to make these standards flexible, extendable, and adaptable, and therefore applicable to additional systems (e.g., experiments with ticks and other vectors, or mechanical transmission components of Chagas disease by triatomine insects).

## Methods

**Fig. 1:**
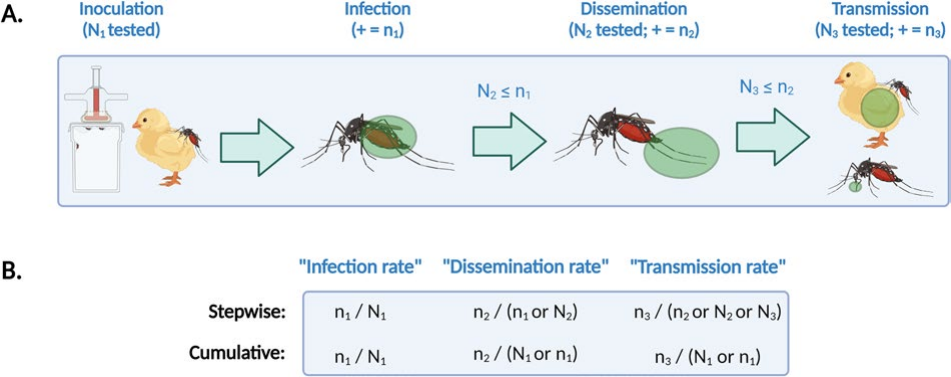
(**A**) For mosquito-borne viruses, vector competence experiments follow a relatively standardized format. Mosquitoes are inoculated with a virus through intrathoracic inoculation or by feeding on a live host or a prepared blood meal; infection and dissemination are measured by testing different mosquitoes tissues; and transmission is measured either by testing saliva or salivary glands, or by allowing mosquitoes to feed on a susceptible host and infect them. (**B**) The results are best understood as rates, but each rate might be reported in several formats; this is further complicated if only a subset of mosquitoes are tested at each stage (e.g., if some mosquitoes die between stages of the experiment). As a result, reporting only denominators leaves much to be desired. Instead–as our data standard reflects–the clearest presentation of raw data is to report total counts of tested and positive mosquitoes at each stage. (“+” indicates how many mosquitoes test positive out of the total sample). Created with BioRender.com.

Tables 1–4 provide a standard checklist for data that arise from, and metadata about, vector competence experiments (and a blank Excel file with these columns is available as Supplementary File 1, for researchers to use directly as a template when reporting primary data along with publications). We have designed these standards with a particular focus on applicability to mosquito-borne arboviruses, and on capturing aspects of experimental design that are known confounders (e.g., rearing and experimental temperature, or inoculation route and dose)^15–19^. While reviewing the literature to design the standard, we found that many of the rates reported (e.g., transmission rate) are derived from discrete and detailed experimental information, yet the original raw data may never be reported, and is often impossible to reconstruct from provided bar or line charts. Moreover, the derived quantities often follow different calculations, with (usually intentional but) very different biological meaning (e.g., the difference between ‘dissemination rate’ and ‘disseminated infection rate’ which are often used interchangeably) (Fig. 1)^20^. Given these choices, it may be misleading to directly compare derived rates across studies. To avoid this problem, we suggest that reporting raw numbers of both vectors tested and those found positive for each basic metric may prevent confusion across study terminology, while still allowing derived rates to be calculated and reported in publications.

**Table 1:** A minimum standard for vector metadata.

Variable	Descriptor
Vector	Full Latin name (species). [Vector taxonomy should be as up-to-date as possible at the time of publication, potentially including additional columns anchored to taxonomic reference databases (e.g., “NCBI:txid7159” is a stable reference for *Aedes aegypti* in the NCBI taxonomic backbone, and will continue to be even if the name changes in the future).]
Vector subsp.	Vector subspecies epithet if applicable (e.g., *formosus*)
Vector strain	Lab reared vector strain name (if one exists)
Vector origin (country)	Wild source for original vector collection (country)
Vector origin (locality)	Wild source for original vector collection (more detailed text string)
Vector origin (year)	Year of wild vector collection
Vector gen.	Generation of vectors in lab colony

**Table 2:** A minimum standard for virus metadata.

Variable	Descriptor
Virus	Virus species name (using appropriate species concept)
Viral lineage	Virus intraspecific lineage (e.g., Asian lineage of Zika virus) if known
Viral strain	Viral strain name (if one exists)
Viral GenBank accession	Accession number for viral sequences
Viral origin (locality)	Where a virus was originally sourced from in humans or wild animals
Viral origin (year)	When a viral strain was sourced from humans or wild animals
Viral history (passage)	Cell type and passage number (e.g., Vero cells, passage 2)
Virus history (freshness)	Was viral stock frozen at any stage in the process, or was it collected fresh and directly used in experiments?

**Table 3:** A minimum standard for experimental metadata.

Variable	Descriptor
Exposure route	How were vectors exposed (e.g., blood meal, live animal, intrathoracic injection)
Host (exp.)	Host species used for live animal or blood meal exposure, and to test transmission, if applicable (either for live animal or for origin of blood meal, if known)
Host lineage/origin	Host intraspecific lineage name, or origin (e.g., if wild-caught), if applicable (either for live animal or for origin of blood meal, if known)
Diagnostic (virem.)	If host viremia was measured in live animal, diagnostic method used
Titer	Host viremia if live animal; concentration of virus for blood meal or intrathoracic inoculation
Units	Units for titer (above; e.g., PFU/ml, FFU/ml, vRNAs/ml, etc.)
Dose	Dose of live virus injected via intrathoracic inoculation, if applicable
Units	Units for dose (above)
Temp. (reared)	Temperature at which vectors are reared (preferably Celsius). [If temperature is not held constant, can be used to report mean, with additional columns added for e.g., “amplitude,” or minimum and maximum temperatures]
Temp. (EIT)	Temperature at which extrinsic incubation happens (preferably Celsius). [If temperature is not held constant, can be used to report mean, with additional columns added for e.g., “amplitude,” or minimum and maximum temperatures]
DPI	Days post-infection (i.e., post-exposure)

**Table 4:** A minimum standard for experimental outcome data.

Variable	Descriptor
Body part (inf.)	Vector tissue or body part used to establish infection (e.g., midgut, whole body, carcass, etc.)
Diagnostic (inf.)	How vector infection was established (e.g., qRT-PCR, virus isolation, etc.)
# tested (inf.)	Number of vectors tested for infection
# inf.	Number of vectors with viral infection
Body part (dissem.)	Vector tissue or body part used to establish dissemination (e.g., legs or wings)
Diagnostic (dissem.)	How viral dissemination in the vector was established (e.g., qRT-PCR, viral isolation, etc.)
# tested (dissem.)	Number of vectors tested for dissemination
# dissem.	Number of vectors with viral dissemination
Host (transm.)	Host species used to test transmission, if tested using live animal exposure to infected vectors
Tissue (transm.)	Tissue (e.g., saliva) used to test ability to transmit (not applicable if live animal exposure and infection was used to test transmission)
Diagnostic (transm.)	How viral transmission in the exposure host (e.g., qRT-PCR, clinical symptoms) or vector tissue (e.g., plaque assay) was established
# tested (transm.)	Number of vectors tested for ability to transmit
# transm.	Number of vectors that were able to transmit

Finally, we note that our goal here is only to provide a minimum standard for even the most basic experiment; more specialized designs may require additional columns, and bespoke solutions to those problems may, as they are developed, become future standardized templates. For example, experimental designs focused on coinfection with additional microbes (e.g., *Wolbachia*; insect-specific viruses, or ISVs) will likely need to reproduce many of the “Virus metadata” variables as a set of “Coinfection metadata” variables, and might also require additional fields. Once standardized, this could be incorporated into a future version of the base template, encouraging more researchers to assay and report the microbes present in laboratory populations, and thereby reducing unquantified heterogeneity among experimental designs.

## Results

**Fig. 2:**
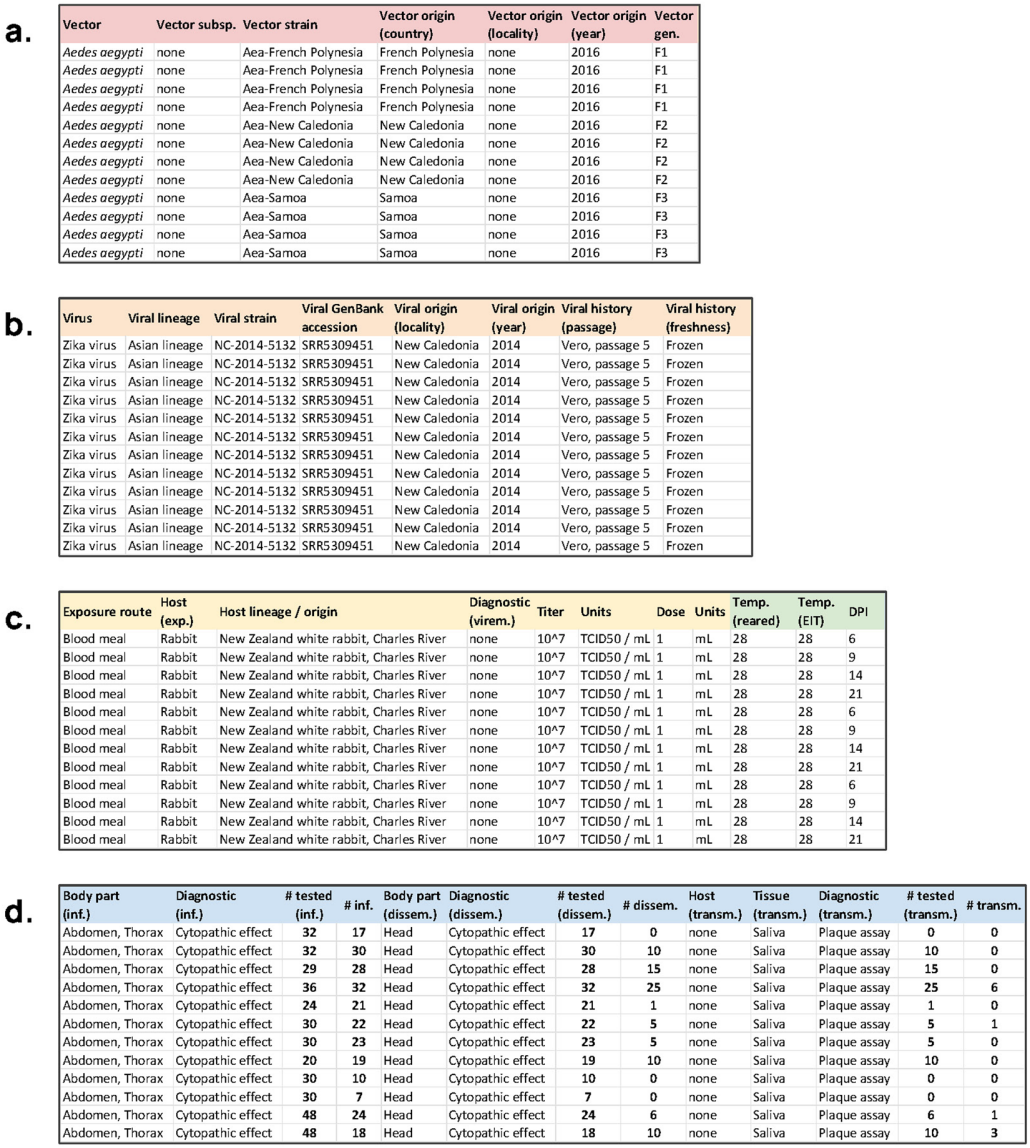
The same dataset (Table 5) in a metadata-complete format with standardized columns, reporting (**a**) ID’s for experimental group, and vector species and vector metadata; (**b**) virus species and viral metadata; (**c**) experimental protocols; and (**d**) the standard results in infection/dissemination/transmission, with clear data on diagnostics and denominators.

To illustrate the data standard in practice, we revisit a study by Calvez *et al*.^21^ of vector competence for Zika virus in *Aedes* mosquitoes relevant to Pacific islands. Unlike many studies, which report results in a mix of summary tables and bar or line graphs, Calvez *et al*. provided very detailed summaries of raw data in their supplementary tables (Table 5). Because they report results in a structured format, with detailed data on the experimental results, other studies have been able to gather their findings alongside other studies (e.g., Table 6). However, these aggregate datasets often lack important dimensions of metadata. To illustrate how researchers might report primary results in the future, we present a metadata-complete version of the results from Calvez *et al*., that meets the minimum data standard we propose, as interpreted from both their Methods and Supplementary Table 1 (Fig. 2)^22^. In rare cases where information was unavailable (e.g., detailed locality information on the origin of mosquitoes), we use “none” to indicate that no data was provided.

**Table 5:** An example dataset from a set of vector competence experiments with *Aedes aegypti* mosquitoes and Zika virus, as reported in Supplementary Table 1 of Calvez *et al*.^21^ Additional details on experimental protocols are provided in the methods section, and the study reports an additional set of experiments with *Aedes polynesiensis* mosquitoes as well (not shown).

		6 dpi	9 dpi	14 dpi	21 dpi
**% of infection (Number of infected bodies**/**number of mosquitoes tested)**	Aea-New Caledonia	88% (21/24)	73% (22/30)	77% (23/30)	95% (19/20)
	Aea-Samoa	33% (10/30)	23% (7/30)	50% (24/48)	38% (18/48)
	Aea-French Polynesia	53% (17/32)	94% (30/32)	97% (28/29)	89% (32/36)
**% of dissemination (Number of infected heads**/**number of infected bodies)**	Aea-New Caledonia	5% (1/21)	23% (5/22)	22% (5/23)	53% (10/19)
	Aea-Samoa	0% (0/10)	0% (0/7)	25% (6/24)	56% (10/18)
	Aea-French Polynesia	0% (0/17)	33% (10/30)	54% (15/28)	78% (25/32)
**% of transmission (Number of infected saliva**/**number of infected heads)**	Aea-New Caledonia	0% (0/1)	20% (1/5)	0% (0/5)	0% (0/10)
	Aea-Samoa	0% (0/0)	0% (0/0)	17% (1/6)	30% (3/10)
	Aea-French Polynesia	0% (0/0)	0% (0/10)	0% (0/15)	24% (6/25)
**% of efficiency (Number of infected saliva**/**number of mosquitoes tested)**	Aea-New Caledonia	0% (0/24)	3% (1/30)	0% (0/30)	0% (0/20)
	Aea-Samoa	0% (0/30)	0% (0/30)	2% (1/48)	6% (3/48)
	Aea-French Polynesia	0% (0/32)	0% (0/32)	0% (0/29)	17% (6/36)

**Table 6:** An example of how the same data (Table 5) could currently be reported in a synthetic format, reproduced in the same format from a table of the results of *Aedes aegypti* vector competence experiments from several studies, assembled by Souza-Neto *et al*.^27^.

Virus	Mosquito origin	Virus genotype and strain	Vector Competence	
			Infection Route, virus dose	Results
ZIKV	French Polynesia	NC-2014-5132, NC	BM, 107 TCID50/mL	IR: 53 at 6 dpi; 94 at 9 dpi; 97 at 14 dpi, 89 at 21 dpi; TR 0 between 6 and 9 dpi; 24 at 21 dpi
	NC [New Caledonia]			IR: 88 at 6 dpi; 73 at 9 dpi; 77 at 14 dpi, 95 at 21 dpi; TR 0 at 6dpi, 3 at 9 dpi, 0 between 14 and 21 dpi
	Samoa			IR: 33 at 6 dpi; 23 at 9 dpi; 50 at 14 dpi, 38 at 21 dpi; TR 0 between 6 and 9 dpi; 17 at 14 dpi and 30 at 21 dpi

## Discussion

Vector competence experiments can have very real-world and urgent applications, informing how health decision-makers assess risks like “Are temperate vectors permissive to a tropical outbreak spreading north?”^23,24^ or “Is an ongoing epizootic likely to spill over into humans?”^25,26^ However, a lack of standardized data reporting is a barrier to reuse and synthesis in this growing field^4^. In turn, current efforts largely remain disconnected from one another, without any central repository that immortalizes these studies’ findings. Some studies have begun to scale this gap: one study compiled a table of results from several dozen studies of *Aedes aegypti* and various arboviruses (see Table 6)^27^. More recently, another study compiled a dataset of 68 experimental studies that tested 111 combinations of Australian mosquitoes and arboviruses, and analyzed biological signals in the aggregated data^28^. These types of efforts are painstaking, requiring substantial manual curation of metadata, and hundreds more experiments are reported in the literature, yet remain unsynthesized due to this barrier.

Going forward, adopting a data reporting standard might make it easier for researchers to share data in reusable formats, and – in doing so – would support the creation of a database following this format. This could also help explain or resolve the issue of why results across studies are inconsistent, especially historical studies and newer studies, which often use newer and more sensitive techniques (e.g., qRT-PCR as compared to PFU). Storing these data in aggregate would facilitate formal meta-analysis and create new opportunities for quantitative modeling. It would also have practical benefits for researchers, assisting them in disseminating their findings, and potentially reducing duplication of research. To that point, a recent synthetic study found that while some combinations (e.g., *Ae. aegypti* and Zika virus) are extremely well studied, over 90% of mosquito-virus pairs might never have been tested experimentally. Standardizing data more broadly might help researchers identify and fill these gaps, simultaneously supporting infectious disease preparedness and fundamental research into the science of the host-virus network^29^.

## Supplementary information

Supplementary information

## Data Availability

All example data and a blank template for reporting are available on Github at github.com/viralemergence/comet-standard.

## Code availability

No code is used in this manuscript.
